# Virtual care for patients with chronic pain and addictions during the COVID-19 pandemic

**DOI:** 10.1080/24740527.2020.1785856

**Published:** 2020-08-25

**Authors:** Karim Mukhida, Joanne Stewart, Reza Mehrpooya, John Fraser

**Affiliations:** Pain Management Unit, Department of Anesthesiology, Pain Management and Perioperative Medicine, Dalhousie University & Nova Scotia Health Authority, Halifax, Nova Scotia, Canada; Department of Family Medicine, Dalhousie University, Halifax, Nova Scotia, Canada

Recent correspondence from Webster et al.^1^ and D’Alessandro et al.^2^ has highlighted the challenges that the COVID-19 pandemic has posed to the provision of chronic pain care. That populations with the combination of chronic pain and a socioeconomically vulnerable or marginalized status have been particularly affected^1–4^ resonates strongly with our local experience managing patients with chronic pain and addictions or opioid use disorder. The pandemic has led to the suspension of all non-urgent ambulatory clinic visits and group sessions at the Queen Elizabeth II Health Sciences Center Pain Management Unit (QE2 PMU) as well as visits to a community-based outreach clinic for patients with chronic pain and addiction comorbidities in Halifax, Nova Scotia, as of March 18, 2020.

As at SickKids,^1^ the QE2 PMU and outreach clinic immediately transitioned to providing virtual care in order to provide continuity of care to patients with addictions or opioid use disorder. To date, virtual care has been provided to 322 patients solely by telephone for a variety of reasons specific to the socioeconomic circumstances of this patient population. For example, although telemedicine has been used to liaise with our patients in rural communities, this has not been possible since community hospitals have stopped providing such services during the pandemic. Many of our patients do not have access to computers, nor adjuncts such as webcams, nor home access to internet services. Some patients do not have home telephones or cell phones and have had to borrow a phone in order to contact the clinics. Other patients have no fixed address. These barriers have posed challenges in providing ongoing care to these patients. In the past, patients could physically return to the clinics to obtain care, but this is no longer possible due to physical distancing and clinic closures. In order to maintain ongoing care during the pandemic, attempts have been made to set up specific dates and times for telephone follow-ups to facilitate patients’ attempts to access a telephone. Because some patients may not have telephones of their own, the issue has been considered of how the QE2 PMU or outreach clinic can best contact patients without breaching patient confidentiality.

Although chronic pain assessments continue to occur in a multidisciplinary way with both a physician and nurse, virtual care has necessitated changes to certain elements of care provision. For example, it has not been possible to perform urine drug screens or urine tandem mass spectrometry that have been used as components of screening for opioid aberrant and addiction behaviors. It has been more challenging to assess nonverbal cues over the telephone, such as drug-related behavioral changes, and to evaluate for stigmata of recent intravenous drug use, such as track marks or infections. Patients have expressed increased anxiety and stress during the pandemic, which they found has affected their chronic pain. Some patients have endorsed using more medication than usual or more than has been prescribed as a manner of coping. This has resulted in some difficult conversations during which prescribers have resisted increasing opioid doses for what appears to be stress-related symptoms. Whereas some patients may have been going to the pharmacy on a frequent basis to pick up opioids or for witnessed dosing, given advice to maintain physical distancing, some patients have been allowed a greater number of days of carries of medications. For others, daily pickups and witnessed dosing have remained the safer option. We continue to liaise with pharmacists about their perspectives regarding aberrant opioid behaviors because they have the opportunity to see patients who continue to pick up their medications.

The COVID-19 pandemic has created challenges for patients with chronic pain and addictions that cannot be fully addressed by virtual care. Some of our patients have lost their jobs and have found that the costs of medications such as buprenorphine/naloxone are now prohibitive. Some of these patients have resorted to purchasing opioids on the street at lower cost. The Addictions Clinic at the Nova Scotia Hospital provided patients with an opportunity to obtain other types of care that are no longer available, including access to a place to sleep for a few hours, snacks, or the hospital library, as well as the opportunity to socialize with other patients.

Some aspects of virtual care for chronic pain and addiction management have been advantageous. Given the public health emphasis on physical distancing, many patients have been anxious to come to the QE2 PMU because of concerns of becoming infected with the novel coronavirus and so have appreciated the opportunity to obtain chronic pain care at least virtually. Finding transportation to get to the QE2 PMU or outreach clinic is challenging for some patients, and obtaining virtual care has therefore helped them avoid logistical and financial travel barriers. Pharmacists taking verbal orders for opioid prescriptions has been convenient for patients, who no longer have to visit the QE2 PMU or outreach clinic to pick up a duplicate opioid prescription. Pharmacies have also been delivering medications to patients at no cost. From the perspectives of the health care providers, the opportunity to interact with patients virtually has enabled some continuity of care. Indeed, it is anticipated that even once the pandemic has subsided to the extent that usual in-person clinic visits are possible, virtual care for patients with chronic pain and addictions will continue in some form.

## References

[1] Webster F, Connoy L, Sud A, Pinto AD, Katz J. Grappling with chronic pain and poverty during the COVID-19 pandemic. Can J Pain. 2020;4(1):125–28. doi:10.1080/24740527.2020.1766855. PMC7951170 33987492

[2] D’Alessandro LN, Brown SC, Campbell F, Ruskin D, Mesaroli G, Makkar M, Stinson JN. Rapid mobilization of a virtual pediatric chronic pain clinic in Canada during the COVID-19 pandemic. Can J Pain. 2020. doi:10.1080/24740527.2020.1771688. PMC7951167 33987495

[3] Eccleston C, Blyth FM, Dear BF, Fisher EA, Keefe FJ, Lynch ME, Palermo TM, Reid MC, de C Williams AC. Managing patients with chronic pain during the COVID-19 outbreak: considerations for the rapid introduction of remotely supported (eHealth) pain management services. Pain. 2020;161(5):889–93. doi:10.1097/j.pain.0000000000001885. 32251203 PMC7172975

[4] Shanthanna H, Strand NH, Provenzano DA, Lobo CA, Eldabe S, Bhatia A, Wegener J, Curtis K, Cohen SP, Narouze S. Caring for patients with pain during the COVID-19 pandemic: consensus recommendations from an international expert panel. Anaesthesia. 2020;75(7):935–44. doi:10.111/anae15076. 32259288 PMC7262200

